# Glucagon-like peptide 1 receptor agonists and the clinical outcomes of inflammatory bowel disease: a systematic review and meta-analysis

**DOI:** 10.1093/ecco-jcc/jjaf181

**Published:** 2025-10-10

**Authors:** Ahmed B Bayoumy, Lindsay M Clarke, Parakkal Deepak, Aakash Desai, Priya Sehgal, uri Gorelik, Haggai Bar-Yoseph, Marie Villumsen, Chris J J Mulder, Dirk J Stenvers, Maarten E Tushuizen, Nanne K H de Boer

**Affiliations:** Department of Gastroenterology and Hepatology, Amsterdam University Medical Center, Amsterdam, The Netherlands; Amsterdam Gastroenterology Endocrinology Metabolism Research Institute, Amsterdam, The Netherlands; Division of Gastroenterology, Hepatology and Endoscopy, Department of Medicine, Harvard Medical School, Brigham and Women’s Hospital, Boston, MA, United States; Division of Gastroenterology and Inflammatory Bowel Diseases Center, Washington University in Saint Louis School of Medicine, St Louis, MI, United States; Division of Gastroenterology, Hepatology, and Nutrition, Allegheny Health Network, Pittsburgh, PA, United States; Division of Gastroenterology and Hepatology, Thomas Jefferson University Hospital, Philadelphia, PA, United States; Department of Internal Medicine, Rambam Health Care Campus, Haifa, Israel; Department of Gastroenterology, Rambam Health Care Campus, Haifa, Israel; The Ruth & Bruce Rappaport Faculty of Medicine, Technion-Israel institute of technology, Haifa, Israel; Center for Clinical Research and Prevention, Bispebjerg and Frederiksberg Hospital, The Capital Region, Copenhagen, Denmark; Department of Gastroenterology and Hepatology, Amsterdam University Medical Center, Amsterdam, The Netherlands; Amsterdam Gastroenterology Endocrinology Metabolism Research Institute, Amsterdam, The Netherlands; Amsterdam Gastroenterology Endocrinology Metabolism Research Institute, Amsterdam, The Netherlands; Department of Endocrinology and Metabolism, Amsterdam University Medical Center, University of Amsterdam, Amsterdam, The Netherlands; Department of Gastroenterology and Hepatology, Leiden University Medical Center, Leiden, The Netherlands; Department of Gastroenterology and Hepatology, Amsterdam University Medical Center, Amsterdam, The Netherlands; Amsterdam Gastroenterology Endocrinology Metabolism Research Institute, Amsterdam, The Netherlands

**Keywords:** GLP1-RA, inflammatory bowel disease, type 2 diabetes mellitus, obesity, Crohn’s disease, ulcerative colitis, hospitalization, corticosteroid use, meta-analysis, systematic review, drug repurposing

## Abstract

**Background:**

Prior studies showed worse outcomes in obese inflammatory bowel disease (IBD) patients, especially those related to hospitalizations, surgery, and steroid-free remission. Glucagon-like peptide-1 receptor agonists (GLP1-RAs) have demonstrated significant metabolic benefits for patients with type 2 diabetes mellitus (T2DM) and obesity. Hence, GLP1-RAs may improve clinical outcomes in patients with IBD, especially those with obesity. The objective was to systematically evaluate the impact of GLP1-RAs on clinical outcomes in patients with IBD.

**Methods:**

A comprehensive literature search was performed using the databases PubMed, Embase, Web of Science, and Cochrane Library from inception to March 15, 2025. Studies reporting outcomes related to GLP1-RAs in patients with IBD were included. Primary outcomes included weight loss and various IBD-related co-endpoints such as hospitalizations, surgery, corticosteroid use, and advanced therapy initiation.

**Findings:**

In total, 11 studies with 16 242 patients with IBD treated with GLP1-RAs were included. Weight loss was achieved using semaglutide (−9.6 kg, 95% confidence interval [CI]: −12.0; −7.2), liraglutide (−9.4 kg, 95% CI: −13.0; −5.8), and tirzepatide (−11.8 kg, 95% CI: −18.3; −5.4) after 3 months of follow-up. In meta-analyses, GLP1-RAs were associated with lower risk of surgery for effect sizes (logHR: 0.61 [95% CI: 0.44-0.84], *I *^2^ = 0%) and event frequencies (odds ratio [OR]: 0.46 [95% CI: 0.32-0.67], *I *^2^ = 42%). Sensitivity analysis for body mass index (BMI) showed a lower risk of hospitalizations and surgery in patients with obesity (BMI ≥ 30).

**Interpretation:**

Patients with IBD and obesity using GLP1-RAs were able to achieve significant weight loss and had lower risks of surgery and hospitalizations. Our findings require confirmation in prospective trials of GLP1-RAs in IBD.

## 1. Introduction

Glucagon-like peptide-1 receptor agonists (GLP1-RAs) have emerged as a transformative class of medications, initially developed for the management of type 2 diabetes mellitus (T2DM). Their primary mechanism involves reducing appetite, enhancing insulin secretion, reducing glucagon release, and slowing gastric emptying, all contributing to improved glycemic control and significant weight loss.^1^ Beyond these metabolic effects, recent research has explored their potential benefits in conditions with underlying inflammation, including inflammatory bowel disease (IBD), which encompasses Crohn’s disease (CD) and ulcerative colitis (UC).^2^ IBD is characterized by chronic inflammation of the gastrointestinal tract, driven by a dysregulated immune response to intestinal microbiota in genetically predisposed individuals. While conventional therapies such as 5-aminosalicylates, corticosteroids, immunosuppressants, and advanced therapies have significantly improved outcomes, they are not without limitations, including incomplete response rates and adverse effects.^3^^,^^4^ Emerging evidence suggests that GLP1-RA may influence pathways relevant to IBD pathophysiology.^5^^,^^6^ In preclinical models, GLP-1 receptor activation has been associated with anti-inflammatory effects, improved intestinal barrier function, and modulation of gut microbiota.^7–9^ Additionally, the metabolic benefits of these agents, such as weight loss and reduced visceral adiposity, may indirectly impact disease activity in IBD, as obesity and metabolic syndrome have been linked to worse outcomes in patients with IBD.^10^^,^^11^ This intersection of metabolic and inflammatory modulation raises the question of whether GLP1-RA could provide therapeutic benefit in IBD. Early observational studies are beginning to shed light on their efficacy and safety in this patient population. One of the safety aspects of GLP1-RAs is the risk of intestinal obstruction which may be caused by delayed intestinal motility affected by GLP1-RAs.^12–14^ Furthermore, on patients with obesity and IBD, previous studies have reported worse outcomes in terms of steroid-free remission, preventable readmissions, and increased risk of surgery.^15–19^ Taken this into account, the primary aim of this study was to synthesize the aggregate data on the effects of GLP1-RAs on the clinical outcomes of IBD.

## 2. Methods

### Study design and registration

This study is a systematic review and meta-analysis, which was conducted following the Preferred Reporting Items for Systematic Reviews and Meta-Analyses (PRISMA) guidelines (Supplementary Material 15).^20^ The study protocol was registered in the OSF Registry with the link https://doi.org/10.17605/OSF.IO/WABE5.

### Research question and framework

The research question was formulated using the patient, intervention, comparator, and outcome (PICO) framework:

Patients (P): Individuals diagnosed with IBD, including CD and UC, who have coexisting obesity and/or T2DM.

Intervention (I): GLP1-RAs.

Comparator (C): Non-GLP-1 therapy or placebo.

Outcomes (O): Relevant clinical outcomes of IBD, including hospitalization rates, corticosteroid initiation, treatment escalation to advanced therapies, and surgical interventions. Secondary outcomes included weight loss and changes in biomarkers (eg, HbA1c, C-reactive protein [CRP], fecal calprotectin [FCP]).

### Literature search strategy

A comprehensive literature search was performed using the databases PubMed, Embase, Web of Science, and Cochrane Library from inception to March 15, 2024. The following keywords and MeSH terms were used: “GLP-1 receptor agonist,” “inflammatory bowel disease,” “Crohn’s disease,” “ulcerative colitis,” “obesity,” “type 2 diabetes,” “hospitalizations,” “remission,” “biomarkers,” and “weight loss.” Boolean operators (AND, OR) and filters (eg, human studies, English language) were applied to refine the search. The search strategy is detailed in Supplementary Material 1.

### Inclusion and exclusion criteria

Inclusion criteria:

- Studies involving patients with IBD and coexisting obesity and/or T2DM.
- Studies assessing the effects of GLP1-RA compared to non-GLP1-RA therapies or placebo.
- Reporting at least 1 primary outcome (eg, hospitalizations, corticosteroid initiation, treatment escalation to advanced therapies [ie, biologicals and/or small molecule therapies], and surgery).
- Clinical trials, observational studies, and cohort studies.

Exclusion criteria:

- Non-human or preclinical studies.
- Conference abstracts.

### Data extraction and quality assessment

Two independent reviewers (A.B. and M.T.) extracted data using a standardized data collection form. Extracted information included study characteristics (eg, author, year, design, and sample size), patient demographics, intervention details (type of GLP1-RA), comparator characteristics, and reported outcomes. Discrepancies were resolved through discussion or consultation with a third reviewer (N.d.B.). Risk of bias in included observational studies was assessed using the ROBINS-I tool (risk of bias in non-randomized studies of interventions) by 2 reviewers (A.B. and M.T.).^21^ ROBINS-I evaluates 7 domains: bias due to confounding, participant selection, classification of interventions, deviations from intended interventions, missing data, measurement of outcomes, and selection of the reported result. Two reviewers independently assessed each study, disagreements were resolved with a third reviewer (N.d.B.).

### Synthesis of evidence

A meta-analysis was conducted using Review Manager (RevMan) software. Both effect sizes (hazard ratios [HRs]) and dichotomous data (odds ratios [ORs]) were analyzed for hospitalizations, surgeries, corticosteroid initiation, and advanced therapy initiation across IBD, CD, and UC cohorts. The definitions of the outcomes of the included studies can be found in Supplementary Material 2. We use both effect sizes and dichotomous data from different studies to assess whether inconsistencies occurred between the different statistical methods. An additional analysis was performed for intestinal obstruction using dichotomous data. The random-effects model was applied because of expected heterogeneity among studies. Vote counting (Table 1) was performed by assessing the direction of effect for each of the different meta-analyses. We deemed a significant finding if half or more of studies showed a similar direction of effect.

**Table 2.** Demographic and study details of included studies.

Authors, year	Population	IBD subtype	T2DM (drugs)	IBD (drugs)	Country	Setting	Outcome	Adjustment
**Clarke,^14^ 2025**	IBD with obesity (*n* = 272)	CD (*n* = 137) UC (*n* = 130)	Semaglutide (*n* = 120) Liraglutide (*n* = 51) Tirzepatide (*n* = 25) Dulaglutide (*n* = 71) Exenatide (*n* = 5)	5-ASA (*n* = 64) Anti-TNF (*n* = 64) IM (*n* = 13) Anti-integrin (*n* = 19) IL12/23 or 23 (*n* = 23) Small molecule (*n* = 3) None (*n* = 64)	USA	Academic hospital	Total weight loss (%), change in BMI, weight loss ≥5%, weight loss ≥10%, IBD flare	Age, baseline BMI, IBD diagnosis, and prior bariatric surgery
**Anderson,^22^ 2024**	IBD with T2DM and/or obesity/MASH (*n* = 120)	CD (*n* = 61) UC (*n* = 59)	Dulaglutide (*n* = 34) Exenatide (*n* = 8) Liraglutide (*n* = 15) Semaglutide (*n* = 90) Tirzepatide (*n* = 27)	Biologicals (*n* = 73)	USA	Academic hospital	Weight loss, IBD-related hospitalizations, IBD clinical scores (HBI and MMS)	N.A.
**Nielsen,^23^ 2024**	IBD with obesity and/or T2DM (*n* = 4430)	CD (*n* = 1500) UC (*n* = 2930)	N.R.	N.R.	Denmark	Nation-wide	Ileus or intestinal obstruction	Age at diagnosis of IBD, sex, type of IBD, steroid use, prior ileus or intestinal obstruction, diabetes status, and small bowel or colon surgery
**Gorelik,^24^ 2024**	IBD with T2DM (*n* = 3737)	CD (*n* = 1854) UC (*n* = 1883)	DPP-4I (*n* = 311) Insulin (*n* = 287) Biguanides (*n* = 1513) SGLT2I (*n* = 110) SU (*n* = 241)	5-ASA (*n* = 1676) OC (*n* = 418) TNF-α (*n* = 131) Thiopurines (*n* = 203) MTX (*n* = 42) UST (*n* = 5) Vedo (*n* = 32)	Israel	Nation-wide	Composite outcomes (steroid dependency, IBD treatment escalation, IBD-related hospitalization, abdominal/perianal surgery, or death)	Age, sex, body mass index, HbA1c, hemoglobin, previous surgery, perianal disease, age at IBD diagnosis, use of biologic therapy or small molecules, sulfonylureas, biguanides, DPP-4I, SGLT2I, and insulin
**St-Pierre,^25^ 2024**	IBD with T2DM (*n* = 36)	CD (*n* = 23) UC (*n* = 12) IBDu (*n* = 1)	Tirzepatide or semaglutide	Advanced therapies (excl. IM): 86%	USA	Academic hospital	Weight loss (BMI), lipid panel changes, FCP and CRP	N.A.
**Levine,^26^ 2024**	IBD with obesity and/or T2DM (*n* = 224)	CD (*n* = 100) UC (*n* = 97) IBDu (*n* = 27)	Semaglutide (*n* = 148) Liraglutide (*n* = 47) Dulaglutide (*n* = 16) Tirzepatide (*n* = 12) Exenatide (*n* = 1)	Corticosteroids (22.3%)	USA	Academic hospital	Hospitalization, corticosteroids prescription, surgery, escalation in advanced therapy (biologicals), CRP, weight loss (BMI), lipid panel	Baseline BMI
**RamosBelinchón,^27^ 2024**	IBD with obesity (*n* = 16)	CD (*n* = 9) UC (*n* = 7)	Liraglutide (*n* = 11) Semaglutide (*n* = 5)	OC (*n* = 3) IM (*n* = 4) TNF-α (*n* = 10) Vedo (*n* = 1)	Spain	Academic hospital	Weight (kg), HBI, partial mayo score, FCP, CRP, lipid panel, creatinine	N.A.
**Desai,^28^ 2024 (1)**	IBD with T2DM (*n* = 2270)	CD (*n* = 1140) UC (*n* = 1130)	Dulaglutide (*n* = 1066) Liraglutide (*n* = 424) Semaglutide (*n* = 1067)	Steroids (*n* = 933) Thioprines (*n* = 302) MTX (*n* = 136) TNF (*n* = 390) UST (*n* = 88) Vedo (*n* = 94)	USA	Multicenter database	Composite outcome includes intravenous steroid use and/or total colectomy	Age, gender, race, nicotine dependence, alcohol-related disorders, primary sclerosing cholangitis, and IBD medications
**Desai,^29^ 2024 (2)**	IBD with obesity (*n* = 150)	CD (*n* = 99) UC (*n* = 51)	Semaglutide (*n* = 150)	5ASA (*n* = 46) Thiopurine (*n* = 14) MTX (*n* = 15) TNF (*n* = 44) Vedo (*n* = 11) UST (*n* = 10) Steroids (*n* = 21)	USA	Multicenter database	Total body weight, oral steroid use, intravenous steroid use, advanced therapy initiation, IBD-related surgery, hospitalization and ED visit	N.A.
**Sehgal,^30^ 2024**	IBD patients with T2DM and/or obesity/MASG (*n* = 244)	CD (*n* = 142) UC (*n* = 102)	Dulaglutide (*n* = 47) Semaglutide (*n* = 131) Liraglutide (*n* = 38) Exenatide (*n* = 3) Tirzepatide (*n* = 5)	5-ASA (*n* = 49) IM (*n* = 8) Biological (*n* = 79) Small molecule (*n* = 4)	USA	Academic hospital	Change in body weight, CRP and FCP	N.A.
**Villumsen,^31^ 2021**	IBD with T2DM (*n* = 3571)	CD (*n* = 960) UC (*n* = 2791)	Unclear. DPP-4 and/or GLP1RA	5-ASA (*n* = 1862) OC (*n* = 1644) TC (*n* = 1208) IM (*n* = 558) TNF-α (*n* = 36) No IBD meds (*n* = 964)	Denmark	Nation-wide	Incidence of hospitalization, surgery, oral corticosteroids, or TNF-α inhibitors	Sex, age, calendar year, IBD severity, and metformin use

Abbreviations: ED, emergency department; IM, immunomodulators; MASH, metabolic dysfunction-associated steatohepatitis; MTX, methotrexate; OC, oral corticosteroids; PSC, primary sclerosing cholangitis; SU, sulfonylureas; TC, topical corticosteroids; UST, ustekinumab; Vedo, vedolizumab.

**Table 3.** Outcomes of included studies.

Authors, year	Hospitalization (95% CI)	Surgery (95% CI)	Corticosteroids initiation (95% CI)	Advanced therapy initiation (95% CI)	Other outcomes (95% CI)
**Clarke,^14^ 2025**	N.A.	N.A.	N.A.	N.A.	Flare 12-month pre (*n* = 29, 16.6%) Flare 12-month post (*n* = 23, 13.1%)
**Anderson,^22^ 2024**	Prior to GLP1 Yes, 10 (10.0%) 12-month post-GLP1 Yes, 10 (10.0%)	N.A.	N.A.	N.A.	HBI prior to GLP1: 3.52 HBI 12-month post-GLP1: 3.18 *P* = 0.61 MMS prior to GLP1: 1.61 MMS 12-months post-GLP1: 1.54 *P* = 0.67
**Nielsen,^23^ 2024**	N.A.	N.A.	N.A.	N.A.	Ileus or intestinal obstruction HR: 0.66 (0.43-1.01) HRa: 0.57 (0.36-0.88) Subanalysis Diabetes HRa: 0.59 (0.32-1.10) No diabetes HRa: 0.48 (0.25-0.93) UC HRa: 0.42 (0.21-0.86) CD HRa: 0.74 (0.42-1.30)
**Gorelik,^24^ 2024**	IBD: HRa 0.74 (0.61-0.91)^*^ UC: HRa 0.63 (0.45-0.90)^*^ CD: HRa 0.82 (0.65-1.05)	IBD: HRa 0.84 (0.47-1.50) UC: HRa 0.74 (0.27-2.07) CD: HRa 0.94 (0.47-1.91)	IBD: HRa 0.66 (0.48-0.99)^*^ UC: HRa 0.75 (0.42-1.35) CD: HRa 0.66 (0.41-1.08)	IBD: HRa 0.82 (0.59-1.17) UC: HRa 0.70 (0.37-1.33) CD: HRa 0.92 (0.61-1.39)	Composite outcome IBD: HRa 0.74 (0.62-0.89)^*^ UC: HRa 0.71 (0.52-0.96)^*^ CD: HRa 0.78 (0.62-0.99)^*^ BMI ≥ 30: HRa 0.61 (0.50-0.77)^*^ BMI < 30: HRa 0.94 (0.67-1.31)
**St-Pierre,^25^ 2024**	N.A.	N.A.	N.A.	N.A.	N.A.
**Levine,^26^ 2024**	Prior to GLP1 Yes: 20 (8.9%) No: 204 (91.1%) 12-month post-GLP1 Yes: 17 (7.6%) No: 207 (8.9%) *P* = 0.70	Prior to GLP1 Yes: 3 (1.3%) No: 221 (98.7%) 12-month post-GLP1 Yes: 4 (1.8%) No: 220 (98.2%) *P* = 1.00	Prior to GLP1 Yes: 50 (22.3%) No: 174 (77.7%) 12-month post-GLP1 Yes: 41 (18.3%) No: 183 (81.7%) *P* = 0.27	Prior to GLP1 Yes: 36 (16.1%) No: 188 (83.9%) 12-month post-GLP1 Yes: 28 (12.5%) No: 196 (87.5%) *P* = 0.34	N.A.
**Ramos Belinchón,^27^ 2024**	N.A.	N.A.	N.A.	N.A.	HBI baseline: 3 (1-5) HBI 6 months: 4 (2.75-4.25) *P* = 0.39 Partial mayo score baseline: 1.29 (0-1) Partial mayo score 6 months: 1.00 (1-2.23) *P* = 0.09
**Desai,^28^ 2024 (1)**	UC group (IV steroids) GLP1: 119 (11.7%) Control: 93 (9.2%) HRa: 1.21 (0.92-1.59) *P* = 0.15 CD group (IV steroids) GLP1: 125 (11.8%) Control: 116 (10.9%) HRa: 1.04 (0.80-1.34) *P* = 0.75	UC group GLP1: < 10 Control: 15 (1.5%) HRa: 0.37 (0.14-0.97) *P* = 0.03^*^ CD group GLP1: 34 (3.2%) Control: 58 (5.4%) HRa: 0.55 (0.36-0.84) *P* = 0.005^*^	UC group GLP1: 331 (32.7%) Control: 284 (28.1%) HRa: 1.12 (0.96-1.31) *P* = 0.14 CD group GLP1: 361 (34.1%) Control: 313 (29.5%) HRa: 1.10 (0.95-1.28) *P* = 0.19	UC group GLP1: 31 (3.6%) Control: 36 (4.2%) HRa: 0.82 (0.51-1.33) *P* = 0.43 CD group GLP1: 50 (6.8%) Control: 39 (5.1%) HRa: 1.31 (0.86-2.00) *P* = 0.19	UC group (Composite outcome) GLP1: 123 (12.1%) Control: 104 (10.2%) HRa: 1.12 (0.86-1.45) *P* = 0.39 CD group GLP1: 154 (14.5%) Control: 121 (14.2%) HRa: 0.97 (0.78-1.22) *P* = 0.85
**Desai,^29^ 2024 (2)**	Semaglutide: 17 (11.9%) Control: 39 (27.4%) aOR: 0.35 (0.19-0.67)	Semaglutide: 0 (0%) Control: 10 (5.9%) -	Oral steroids Semaglutide: 38 (26.7%) Control: 44 (30.9%) aOR: 0.81 (0.48-1.36) Intravenous steroids Semaglutide: 10 (7.0%) Control: 14 (9.8%) aOR: 0.69 (0.29-1.61)	Semaglutide: 10 (10.3%) Control: 10 (10.0%) aOR: 1.03 (0.41-2.60)	Any-cause emergency department visit Semaglutide: aOR: 0.92 (0.52-1.61)
**Sehgal,^30^ 2024**	N.A.	N.A.	N.A.	N.A.	N.A.
**Villumsen,^31^ 2021**	GLP1RA and/or DPP4: 178 events, 2.889 PY, (IR per 1000 PY, 61.6). No GLP1RA and/or DPP4: 1445 events, 14.024 PY (IR per 1000 PY 103.0). Crude IRR: 0.60 (0.51-0.70)^*^ Adjusted IRR: 0.73 (0.58-0.91)^*^	GLP1RA and/or DPP4: 97 events, 3.675 PY, (IR per 1000 PY, 26.4). No GLP1RA and/or DPP4: 593 events, 17.456 PY (IR per 1000 PY 34.0). Crude IRR: 0.78 (0.63-0.96)^*^ Adjusted IRR: 0.79 (0.57-1.09)	GLP1RA and/or DPP4: 133 events, 2.813 PY, (IR per 1000 PY, 47.3). No GLP1RA and/or DPP4: 1238 events, 13.104 PY (IR per 1000 PY 94.5). Crude IRR: 0.50 (0.42-0.60)^*^ Adjusted IRR: 0.54 (0.41-0.70)^*^	GLP1RA and/or DPP4: 29 events, 4.183 PY, (IR per 1000 PY, 6.9). No GLP1RA and/or DPP4: 213 events, 18.737 PY (IR per 1000 PY 11.4). Crude IRR: 0.61 (0.41-0.90)^*^ Adjusted IRR: 0.56 (0.32-1.00)	N.A.

* = statistically significant. Abbreviations: CD, Crohn’s disease; HBI, Harvey-Bradshaw index (HBI < 5 is remission); HRA, adjusted hazard ratio; IBD, inflammatory bowel disease; IR, incidence rate; IRR, incidence rate ratio; MMS, modified mayo score; PY, patient-year; UC, ulcerative colitis.

**Table 1.** Summary of outcomes of different meta-analyses performed in this study.

Analysis	Hospitalizations	Surgery	Steroid initiation	Advanced therapies initiation
**I nflammatory bowel disease (IBD) (effect sizes)**	=	↓	=	=
**IBD (event frequencies)**	=	↓	=	=
**Ulcerative colitis (effect sizes)**	=	=	=	=
**Crohn’s disease (effect sizes)**	=	=	=	=
**Body mass index (BMI) ≥ 30**	↓	↓	=	=
**BMI < 30**	↓	=	↓	=
**Summary**	↓ in 2 of 6 analyses No difference in 4 of 6 analyses.	↓ in 3 of 6 analyses No difference in 3 of 6 analyses.	↓ in 1 of 8 analyses No difference in 5 of 6 analyses	No difference in 6 of 6 analyses

### Heterogeneity and publication bias assessment

Heterogeneity was assessed using the *I*^2^ statistic, with values of 25%, 50%, and 75% representing low, moderate, and high heterogeneity, respectively. Funnel plots were used to visually inspect potential publication bias, and Egger’s regression test was performed where applicable.

### Sensitivity and statistical analyses

Sensitivity analyses were performed based on IBD subtype (CD or UC), BMI and GLP1-RA drug (for weight loss). Other subgroup analysis such as presence of T2DM, and visceral adipose tissue were not possible due to lack of data. All statistical analyses were performed using RevMan. A *P*-value of 0.05 was considered statistically significant.

### Ethical considerations

Ethical approval was not required for this study as it involved the synthesis of previously published data.

## 3. Results

### Study and population characteristics

**Figure 1.**
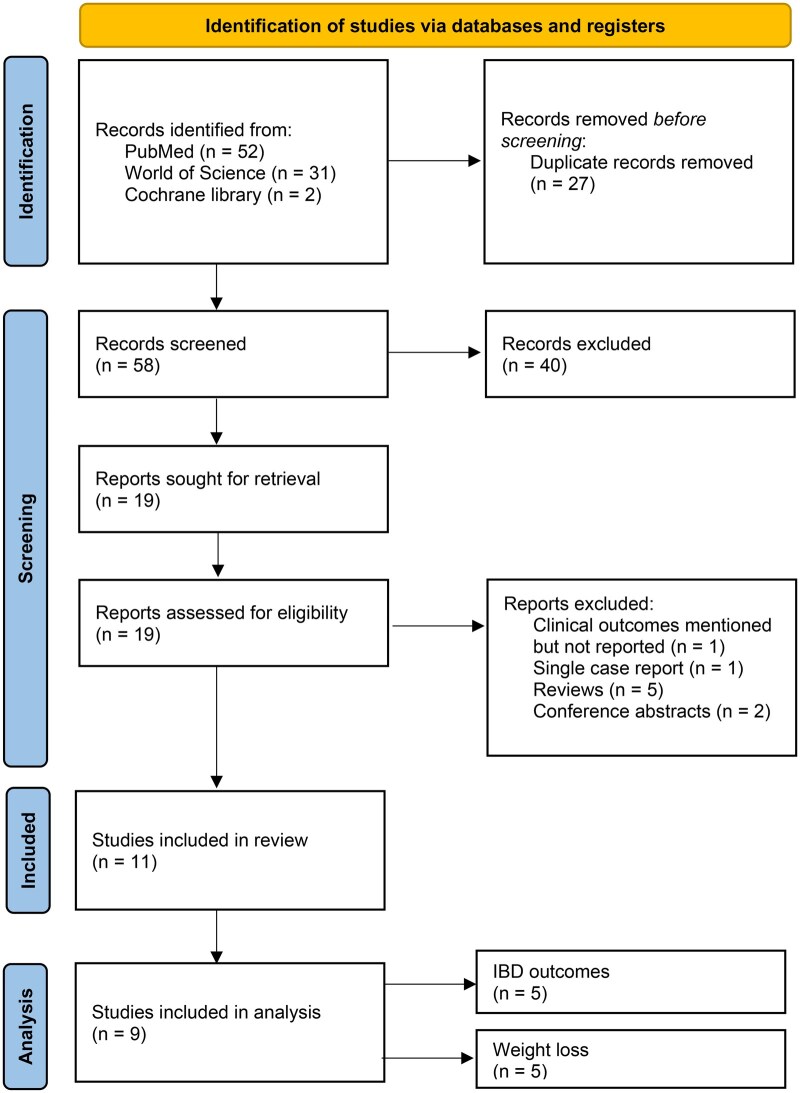
Flowchart of included studies (*n* = 11).

**Figure 2.**
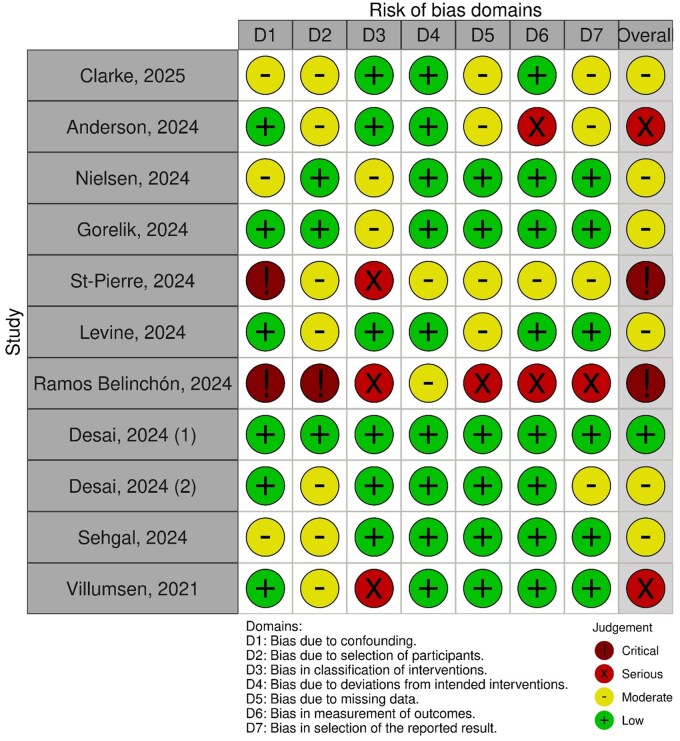
Risk of bias traffic-light plot (created using RobVis-tool).

**Figure 3.**
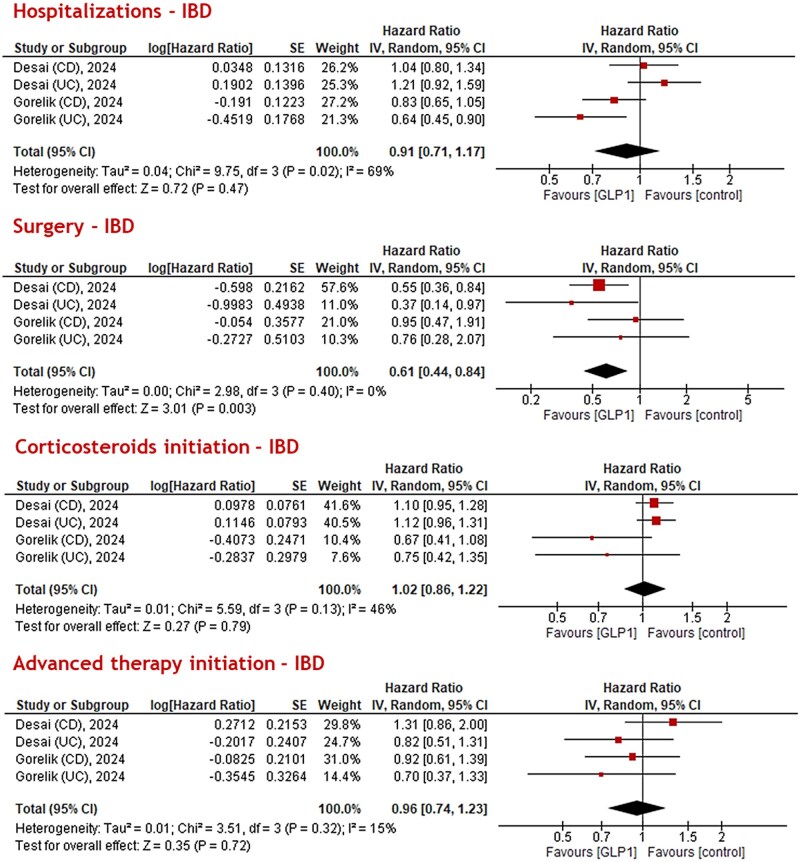
Meta-analysis of different inflammatory bowel disease (IBD)-related outcomes (hospitalizations, surgery, corticosteroids initiation, and advanced therapy initiation) in various studies patients based on published effect sizes (hazard ratio [HR]). Glucagon-like peptide-1 (GLP1) therapy seemed to reduce the risk of surgery (logHR: 0.61, 95% confidence interval [CI]: 0.44-0.84, *P* = 0.003, *I *^2^ = 0%).

**Figure 4.**
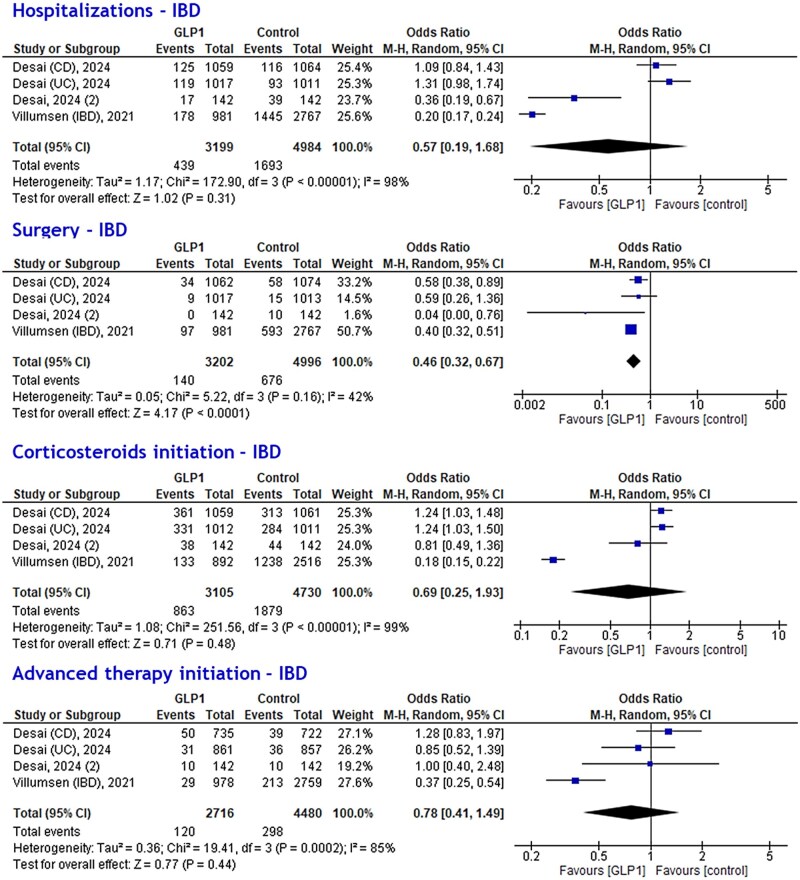
Meta-analysis of different inflammatory bowel disease (IBD)-related outcomes (hospitalizations, surgery, corticosteroids initiation, and advanced therapy initiation) in various studies with IBD patients based on event frequencies (event per patient). meta-analyses showed that Glucagon-like peptide-1 receptor agonist (GLP1-RA) therapy reduced event rates for surgery (odds ratio [OR]: 0.46, 95% confidence interval [CI]: 0.32-0.67, *P* < 0.0001).

**Figure 5.**
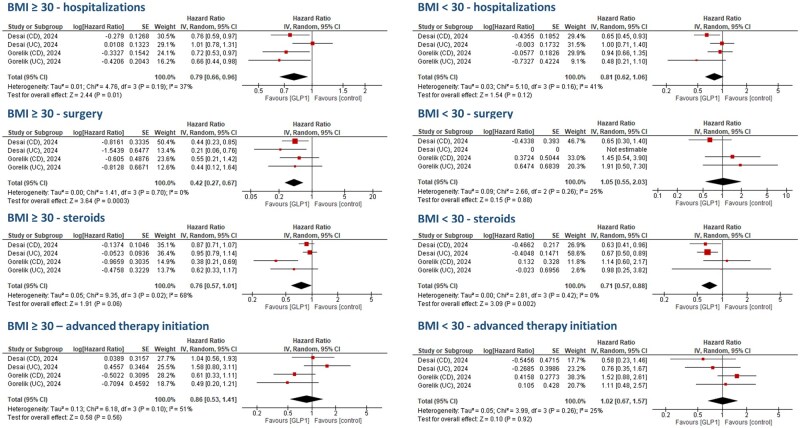
Sensitivity analyses for body mass index (BMI) for each of the clinical outcomes. In inflammatory bowel disease (IBD) patients with BMI ≥ 30 glucagon-like peptide-1 receptor agonist (GLP1-RA) users had lower risks of hospitalizations (logHR: 0.79, 95% confidence interval [CI]: 0.66-0.96, *I*^2^ = 37%, *P* = 0.01) and surgery (logHR: 0.42, 95% CI: 0.27-0.67, *I*^2^ = 0%, *P* = 0.0003) compared to non-GLP1-RA users, while in IBD patients with BMI < 30 (logHR: 0.71, 95% CI: 0.57-0.88, *I*^2^ = 0%, *P* = 0.002) GLP1-RA users had lower risk for steroid initiation.

**Figure 6.**
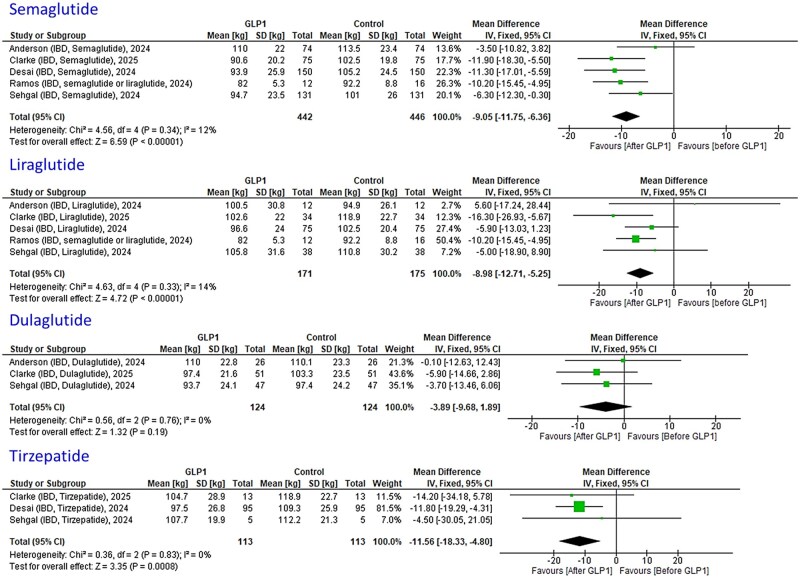
Meta-analysis of weight loss in inflammatory bowel disease (IBD) patients using different glucagon-like peptide-1 receptor agonist (GLP1-RA) therapies. All GLP1-RAs (semaglutide, liraglutide, and tirzepatide), expect one GLP1-RA (dulaglutide), showed statistical significant weight loss in IBD patients.

**Figure 7.**
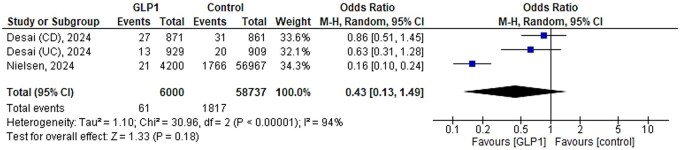
Meta-analysis of intestinal obstruction in various studies based on the reported number of patients. The odds-ratio’s (OR) were calculated, and a random-effects model was used for the analysis. There was no clear difference in intestinal obstruction between glucagon-like peptide-1 receptor agonist (GLP1-RA) therapy and controls, although the point estimate suggests lower event rates for GLP1-RAs compared to controls (OR: 0.43, 95% confidence interval [CI]: 0.13-1.49, *I*^2^ = 94%, *P* = 0.18).

This systematic review included 11 observational studies ­(Figure 1) evaluating the effects of GLP1-RAs on clinical outcomes in patients with IBD complicated by T2DM and/or obesity. A total of 16 242 patients with IBD using GLP1-RAs were analyzed, with representation from both CD and UC cohorts (Table 2). The meta-analyses from those patients can be found in Figures 2-7 and Supplementary Materials 3-5. The studies were conducted across diverse settings, including academic hospitals in the United States, Spain, and Israel, as well as nationwide registries in Denmark, Israel, and the United States (Table 2). The summary of clinical and metabolic outcomes can be found in Tables 1 and 3. The results of the risk of bias assessments can be found in Figure 2.

### Hospitalization rates

The impact of GLP1-RAs on hospitalization rates for IBD was similar to controls. The meta-analysis using effect sizes showed that GLP1-RA therapy lead to similar rates of hospitalizations in patients with IBD compared to non-GLP1-RA therapy (HR: 0.91, 95% confidence interval [CI]: 0.71-1.17, *I*^2^ = 69%, *P* = 0.47) (Figure 3). Sensitivity analysis for UC (HR: 0.88, 95% CI: 0.66-1.18, *I*^2^ = 51%) and CD (HR: 0.93, 95% CI: 0.78-1.10, *I*^2^ = 0%) showed similar trends (Supplementary Materials 3 and 4). In the meta-analysis using event frequencies, similar hospitalization events in the GLP1-RA group (OR: 0.57, 95% CI: 0.19-1.68, *I*^2^ = 98%) (Figure 4) compared to controls were found. Sensitivity analysis (Figure 5) for BMI revealed that patients with BMI ≥ 30 and GLP1-RA therapy (logHR: 0.79, 95% CI: 0.66-0.96, *I*^2^ = 37%, *P* = 0.01) had reduced risks of hospitalization. For patients with BMI < 30 and GLP1-RA therapy, the point estimate also suggested reduced risk compared to no GLP1-RA therapy and BMI <30 (logHR: 0.81), but the 95% CI (0.62-1.06) included 1.0, indicating the result was not statistically significant (*I*^2^ = 41%; *P* = 0.12).

### Surgical interventions

The potential impact of GLP1-RAs on the risk of surgery was positive. In meta-analysis, the pooled HR for surgery was in favor of GLP1-RA therapy compared to non-GLP1-RA therapy (logHR: 0.61, 95% CI: 0.44-0.84, *I*^2^ = 0%, *P* = 0.003) in patients with IBD (Figure 3**)**. No differences were found in the sensitivity analysis for UC (logHR: 0.79, 95% CI: 0.43-1.45, *I*^2^ = 0%, *P* = 0.44), and for CD (logHR: 0.68, 95% CI: 0.37-1.26, *I*^2^ = 53%, *P* = 0.22) (Supplementary Materials 3 and 4). Using event frequencies, surgery events were significantly lower for the GLP1-RA group in patients with IBD (OR: 0.46, 95% CI: 0.32-0.67, *I*^2^ = 42%) (Figure 4). Sensitivity analysis (Figure 5) for BMI showed that patients with BMI ≥ 30 and GLP1-RA therapy had reduced risk for surgery (logHR: 0.42, 95% CI: 0.27-0.67, *I*^2^ = 0%, *P* = 0.0003), while patients with BMI < 30 and GLP1-RA therapy had no reduced risk for surgery compared to no GLP-RA therapy and BMI < 30 (logHR: 1.05, 95% CI: 0.55-2.03, *I*^2^ = 25%, *P* = 0.88).

### Corticosteroid initiation

The effect of GLP1-RAs on corticosteroid initiation varied across studies. Meta-analysis showed no effect of GLP1-RA on corticosteroid initiation in patients with IBD (logHR 1.02, 95% CI: 0.86-1.22, *I*^2^ = 46%, *P* = 0.79) (Figure 3). Similar results were obtained in the sensitivity analysis for UC (logHR 1.02, 95% CI: 0.73-1.42, *I*^2^ = 40%, *P* = 0.91) and CD (logHR 0.90, 95% CI: 0.56-1.47, *I*^2^ = 74%, *P* = 0.68) (Supplementary Materials 3 and 4). Meta-analyses using event frequencies showed that corticosteroid initiation was not different between GLP1-RA and control groups among patients with IBD (OR: 0.69, 95% CI: 0.25-1.93, *I*^2^ = 99%, *P* = 0.48) (Figure 4). Sensitivity analysis (Figure 5) for BMI showed that in patients with BMI < 30 and GLP1-RA therapy there was lower risk of corticosteroid initiation (logHR: 0.71, 95% CI: 0.57-0.88, *I*^2^ = 0%, *P* = 0.002). The point estimate also indicated lower risk for corticosteroid initiation in patients with BMI ≥ 30 and GLP1-RA therapy, although this was not statistically significant due to broad confidence intervals (logHR: 0.76, 95% CI: 0.57-1.01, *I*^2^ = 68%, *P* = 0.06).

### Advanced therapy initiation

Meta-analysis showed no effect of GLP1-RA on advanced therapy initiation (ie, biologicals and/or small molecule therapies) in IBD (logHR: 0.96, 95% CI: 0.74-1.23, *I*^2^ = 15%, *P* = 0.72) ­(Figure 3). Advanced therapy initiation did not differ for UC (logHR: 0.78, 95% CI: 0.53-1.14, *I*^2^ = 0%, *P* = 0.20), and CD (logHR 1.10 [95% CI: 0.77-1.55], *I*^2^ = 28%, *P* = 0.61) (Supplementary Materials 3 and 4). The event frequency meta-analysis showed that patients with IBD treated with GLP1-RAs had similar advanced therapy initiation (OR: 0.78, 95% CI: 0.41-1.49, *I*^2^ = 85%, *P* = 0.44) events compared to non-GLP1-RA treatment (Figure 4**)**. Sensitivity analysis (Figure 5) for BMI did not show any difference between GLP1-RAs and controls for patients with BMI ≥ 30 (logHR: 0.86, 95% CI: 0.53-1.41, *P* = 0.56) and BMI < 30 (logHR: 1.02, 95% CI: 0.67-1.57, *P* = 0.92).

### Weight change

Meta-analyses were performed for different GLP1-RA therapies (semaglutide, liraglutide, dulaglutide, and tirzepatide) for weight loss in IBD patients (Figure 6). Statistical significant weight loss was achieved in IBD patients using semaglutide (−9.1 kg, 95% CI: −11.8; −6.4, *I*^2^ = 12%, *P* < 0.00001, *n* = 442), liraglutide (−9.0 kg, 95% CI: −12.7; −5.3, *I*^2^ = 14%, P < 0.00001, *n* = 171), and tirzepatide (−11.6 kg, 95% CI: −18.3; −4.8, *I*^2^ = 0%, *P* = 0.0008, *n* = 113). No statistical significant weight loss was achieved using dulaglutide (−3.9 kg, 95% CI: −9.7; −1.9, *I*^2^ = 0%, *P* = 0.19, *n* = 124). No meta-analysis was performed for exenatide, although 3 studies reported on exenatide, the study by Clarke et al.^14^ only had 1 patient with follow-up. Furthermore, Levine et al.^26^ showed that weight loss could be achieved after 12 months of follow-up in IBD patients with T2DM (BMI 33.5, 95% CI: 29.9-37.0 baseline vs. BMI 31.5, 95% CI: 27.1-35.8 follow-up, *P* < 0.01) and without T2DM (BMI 33.2, 95% CI: 29.3-38.4 baseline vs. BMI 32.6, 95% CI: 28.6-37.5 follow-up, *P* = 0.04), and that significant weight loss could only be achieved in patients with obesity (BMI ≥ 30).

### Intestinal obstruction

Intestinal obstruction has been reported in 3 studies in relation to GLP1-RA use.^23^^,^^28^^,^^32^ The meta-analysis (Figure 7) showed that in these 3 included studies, the number of events was 286 (3.0%) and 2091 (3.4%) in exposed (*n* = 9571) and non-exposed groups (*n* = 62249), respectively. The OR derived from the meta-analysis for intestinal obstruction was not different between GLP1-RA therapy and controls (OR: 0.51, 95% CI: 0.21-1.23), (*I*^2^ = 95%, *P* = 0.13).

### Summary of meta-analyses

Table 1 shows the summary of the meta-analyses for the different IBD-related outcomes. Vote counting was used for each of the individual analyses to determine the direction of the outcome. For hospitalizations, 2 out of 6 analyses were in favor of GLP1-RA therapy, while the other 4 analyses did not show any difference between GLP1-RA and controls. Vote counting for surgery showed that 3 out of 6 were in favor of GLP1-RA therapy. The majority of studies did not show any difference between GLP1-RA and controls for corticosteroid initiation (5 of 6) and advanced therapy initiation (6 of 6).

### Disease activity scores

The effect of GLP1-RAs on disease activity scores and biomarkers was mixed. Anderson et al.^22^ reported no significant improvement in disease activity scores over 12 months, with only marginal reductions in the Harvey–Bradshaw Index (HBI) and Mayo Score (HBI: 3.52-3.18, *P* = 0.61; Mayo: 1.61-1.54, *P* = 0.67). Similarly, Ramos Belinchón et al.^27^ found no significant changes in HBI or partial Mayo scores after 6 months of GLP1-RA treatment.

### C-reactive protein

Inflammatory markers, particularly CRP, showed variable responses to GLP1-RA therapy (Supplementary Material 5). Anderson et al.^22^ observed a significant reduction in CRP levels from 12.9 mg/L at baseline to 6.4 mg/L after 12 months (*P* = 0.005), suggesting a potential anti-inflammatory effect of GLP1-RAs. Conversely, St-Pierre et al. reported no significant change in CRP levels, with median values remaining stable at 3 mg/L (interquartile range [IQR] 3-6.5) throughout the study period (*P* = 0.21). Levine et al.^26^ observed a modest reduction in CRP levels in patients with IBD, from 6.9 mg/L (IQR: 3.1-11.4) to 5.5 mg/L (IQR: 2.8-11.6), though statistical significance was not reported.

### Fecal calprotectin

FCP, a biomarker of intestinal inflammation, showed mixed results across studies (Supplementary Material 5). Ramos Belinchón et al.^27^ reported no significant change in FCP levels after 6 months of therapy, with median levels decreasing slightly from 41.0 µg/g (IQR: 14-80) to 32.7 µg/g (IQR: 13.8-51.0) (*P* = 0.74). Sehgal et al.^30^ observed a decrease in mean FCP levels from 896 µg/g (SD: 2600) at baseline to 705 µg/g (SD 2297) after 12-24 weeks, though the variability in measurements was high. Desai et al.^28^ found no significant differences in mean FCP levels between GLP1-RA users and controls in either the UC (324.7 ± 533 vs. 369.7 ± 624, *P* = 0.67) or CD groups (260 ± 391 vs. 324 ± 487, *P* = 0.29).

### HbA1c levels

Glycemic control, as measured by HbA1c, improved significantly in a few studies (Supplementary Material 5). No meta-analysis was performed because it was unclear which GLP1-RA caused the decrease in HbA1c value. Levine et al.^26^ reported a decrease in HbA1c from 6.5% (IQR: 5.9-7.4) at baseline to 6.2% (IQR: 5.4-7.0) after 12 months of therapy in patients with IBD (*P* = 0.01). Similarly, patients without IBD in the same cohort experienced a comparable reduction. Ramos Belinchón et al.,^27^ however, reported no significant change in HbA1c levels, with a reduction from 6.2% (IQR: 5.5-7.8) to 5.9% (IQR: 5.3-7.58) (*P* = 0.14), suggesting variability in glycemic outcomes across studies.

## 4. Discussion

This systematic review with meta-analysis highlights the potential role of GLP1-RAs in improving clinical outcomes in patients with IBD and obesity and/or T2DM. Our meta-analysis showed that GLP1-RAs significantly reduces weight and reduces risks of hospitalizations and surgery in patients with IBD. It must be noted that effects of GLP1-RAs were heterogenous on other clinical outcomes.

### The role of obesity and visceral adipose tissue in IBD patients

Obesity has been recognized as a risk factor for hospitalizations in patients with IBD. In 1 nationwide cohort study, 5128 obese patients with IBD were matched to 5128 nonobese IBD patients using propensity score matching. Obese patients with IBD were more likely to have preventable readmissions compared to nonobese patients with IBD (19% vs. 15%), and obese patients with IBD spend more days in the hospital annually (median: 8 vs. 5 days, *P* < 0.01) with higher hospitalization-related costs (median: $17 277 vs. $11 847, *P* < 0.01).^16^ In patients with IBD that were hospitalized, obesity was also associated with longer duration of stay (mean: 5.5 vs. 4.9 days, *P* < 0.001) and mean hospital costs ($50 126 vs. $45 001, *P* < 0.001).^33^ Patients with IBD and obesity had lower steroids-free clinical remission at week 24 in a prospective registry study of patients starting with new IBD treatment in regular care in the Netherlands.^15^ In UC patients, it was reported that each increase of BMI (1 kg/m^2^) was associated with 4% increase in the risk of treatment failure (aHR: 1.04, 95% CI: 1.00-1.08) in patients treated with biologicals and 8% increase in the risk of surgery/hospitalization (aHR: 1.08, 95% CI: 1.02-1.14).^34^ In this systematic review, GLP1-RAs were found to reduce weight in our meta-analysis by approximately 10 kg (∼TBW 10%) after >3-6 months of follow-up in IBD patients treated with semaglutide, liraglutide, or tirzepatide. Furthermore, GLP1-RAs reduced the risks of hospitalizations and surgery, in patients with IBD and obesity. We would like to emphasize that BMI alone has been described as an independent risk factor for surgery and hospitalization.^16^^,^^18^ Our findings suggest that GLP1-RA may reduce these risks in patients with IBD and BMI ≥ 30. This is specifically relevant considering higher rates of medical complications during hospitalization and higher financial costs for admission in patients with obesity.

In this study, GLP1-RAs did not reduce advanced therapies or corticosteroid initiation, although sensitivity analysis showed benefits of GLP1-RAs in reducing corticosteroid initiation in patients with and without obesity. Yarur et al.^19^ showed that poor response to biological therapy (infliximab, vedolizumab, or ustekinumab) was associated with a higher intra-abdominal visceral adipose tissue compartment as a percent of total body mass. Similarly, He et al.^35^ showed that non-responders to corticosteroid therapy had higher visceral adipose tissue compared to responders in CD (21.3 vs. 11.6 cm^2^/m^2^, *P* = 0.004). Although for patients with UC, non-responders had lower visceral adipose tissue compared to responders (14.7 vs. 24.4 cm^2^/m^2^, *P* = 0.001). In this systematic review, abdominal visceral fat was not determined in the included studies, hence there is a need for more studies that assess the effects of GLP1-RAs on abdominal visceral fat in relation to corticosteroid and biological therapies.

### Intestinal obstruction and ileus

The meta-analysis showed that patients using GLP1-RA had similar rates of intestinal obstruction compared to non-GLP1-RA therapies (1.0% [*n* = 61] vs. 3.1% [*n* = 1,817]). GLP1-RAs were thought to be associated with higher risks of intestinal obstruction or ileus due to its effects on transit times and intestinal motility due to GLP-1-induced increased intestinal length and villus height.^36^^,^^37^ Sodhi et al.^38^ reported that GLP1-RAs were associated with increased risks of bowel obstruction (HR: 4.22, 95% CI: 1.02-17.4) compared to bupropion-naltrexone in patients with obesity. However, a Danish study by Ueda et al.^39^ in patients with T2DM using GLP1-RAs showed no increased risk of intestinal obstruction (HR: 0.83, 95% CI: 0.69-1.01). The data presented by Ueda et al.^39^ suggests that the risks of intestinal obstruction in patients with IBD using GLP1-RA are not increased, which is similar to the finding of this systematic review.

### Limitations and future directions

This systematic review with meta-analysis has several limitations. The included studies were heterogeneous in design, population characteristics, and outcomes which make it challenging to draw consistent conclusions. Most studies were observational or retrospective, introducing unmeasured potential biases and limiting causal inferences. Furthermore, studies had missing data, did not report clinical outcomes, clinical scores, or BMI/visceral adiposity and data on side-effects were lacking. RCTs are needed to confirm the potential efficacy and safety of GLP1-RAs in IBD. While weight loss and metabolic benefits were consistently observed, the impact of BMI or visceral adiposity on IBD-specific outcomes were not reported consistently. The meta-analyses yielded heterogenous and inconsistent results across other clinical outcomes and sensitivity analyses (eg, BMI and IBD-type). Meta-analyses conducted in this study exhibited moderate to high heterogeneity, suggesting variability in patient populations and reported outcomes. It is important to note that the studies by Desai et al.,^28^ Niu et al.,^32^ and Alchirazi et al.^40^ were performed using the triNetX database. Considering that the studies by Niu et al.^32^ and Alchirazi et al.^40^ were conference abstracts and may have included patient populations overlapping with those reported by Desai et al.,^28^ these studies were not included. Another limitation is that most studies did not report or account for BMI or a measurement of the visceral adipose tissue compartment, which may significantly affect the results. Future research should prioritize RCTs evaluating GLP1-RAs in well-defined IBD populations, stratified by subtype, obesity, and metabolic status. Studies should also assess long-term outcomes, including durability of response, side effects, and explore mechanistic pathways to better understand how GLP1-RAs may influence IBD pathophysiology. Additionally, the potential synergistic effects of combining GLP1-RAs with existing therapies warrant investigation.

### Obesity first approach

Unaddressed issues in this review are the side-effects associated with GLP1-RA, including constipation, gastroparesis, and pancreatitis,^41^ and potential drug interactions with other medications like warfarin and thiopurines.^42^^,^^43^ A shift toward an “obesity first” approach could reshape IBD care by prioritizing obesity as a key underlying factor. By 2030, we anticipate gaining clearer insights into whether GLP-1 therapies will become a long-term treatment option for obesity and potentially for IBD as well. Currently, 2 clinical trials under investigation for GLP1-RAs combined with mirikizumab in IBD.^13^^,^^44^ While the current high cost of these medications limits accessibility and research, the introduction of generics may improve availability and affordability.

Generic pharmaceutical companies are expected to significantly scale up production once patents expire, which also depends on patent and regulatory exclusivities.^45^ However, the future landscape remains uncertain, as it is difficult to predict how other pharmaceutical companies will innovate within the GLP-1 space, whether through combination therapies or other advancements.

## 5. Conclusion

Meta-analysis showed that GLP1-RAs were associated with significant weight loss, and surgery in patients with IBD, and with reduced risk of hospitalization in the subset of obese patients using GLP1-RAs. However, meta- and sensitivity analyses had heterogenous results, hence more research is needed to verify the results with a larger focus on stratification by BMI and visceral adiposity. Cost-effectiveness analysis of GLP1-RAs are necessary especially in the context of IBD-related surgery and hospitalizations. There is a need for randomized studies for IBD patients using GLP1-RA compared with active comparators, focusing both on efficacy and safety.

## Supplementary Material

jjaf181_Supplementary_Data

## Data Availability

All additional data from individual authors are available in the Supplementary Material. The original meta-analysis files can be obtained from the corresponding author by request.
