# Supplementary material for: Glucagon-like peptide 1 receptor agonists and the clinical outcomes of inflammatory bowel disease: a systematic review and meta-analysis
Source: J Crohns Colitis. 2025 Oct 10;19(10):jjaf181. doi: 10.1093/ecco-jcc/jjaf181 (PMC12668684; doi:10.1093/ecco-jcc/jjaf181)
Supplement: jjaf181_Supplementary_Data [file jjaf181_supplementary_data.docx]

**Supplementary material**

**Supplement 1.** Full search terms queries.

**PubMed**

('glucagon-like peptide 1 receptor agonist'/exp OR 'glp-1 receptor agonist' OR 'GLP1-RA' OR 'semaglutide' OR 'liraglutide' OR 'dulaglutide' OR 'exenatide' OR 'tirzepatide')

AND ('inflammatory bowel disease'/exp OR 'crohn disease'/exp OR 'ulcerative colitis'/exp OR 'ibd' OR 'crohn disease' OR 'uc')

**Web of Science**

TS=("glucagon-like peptide 1 receptor agonist" OR "GLP-1 receptor agonist" OR "GLP1-RA" OR "semaglutide" OR "liraglutide" OR "dulaglutide" OR "exenatide" OR "tirzepatide")

AND TS=("inflammatory bowel disease" OR "IBD" OR "Crohn's disease" OR "Crohn disease" OR "ulcerative colitis" OR "UC")

**Cochrane Library**

("glucagon-like peptide 1 receptor agonist" OR "GLP-1 receptor agonist" OR "GLP1-RA" OR "semaglutide" OR "liraglutide" OR "dulaglutide" OR "exenatide" OR "tirzepatide")

AND ("inflammatory bowel disease" OR "IBD" OR "Crohn's disease" OR "Crohn disease" OR "ulcerative colitis" OR "UC")

**Supplement 2. Definitions of parameters and outcomes in the included studies.**

| Author, year | Obesity/diabetes | Hospitalization | Surgery | Steroid use | Advanced therapy | Primary outcome |
| --- | --- | --- | --- | --- | --- | --- |
| Clarke^14^, 2025 | BMI ≥ 30 | See primary outcome | See primary outcome | See primary outcome | See primary outcome | Weight loss of ≥ 5% baseline body weight at 12-months post GLP1-RA initiation, and IBD flare within 12-months after initiation of GLP1-RA. IBD flare was defined as a dichotomous composite outcome of IBD-related ED visits or hospitalizations, surgeries, escalation/change of IBD-directed therapy or steroid use, and compared between the 12-months pre- and post-GLP1-RA therapy. |
| Anderson^26^, 2024 | N/A | IBD  related hospitalizations | N/A | N/A | N/A | Clinical severity scores (Harvey Bradshaw Index for CD and Modified Mayo score for UC), endoscopic scores (Simple Endoscopic Score for CD and the Mayo Endoscopic score for UC), number of IBD-related hospitalizations, and changes in inflammatory marker CRP levels. |
| Nielsen^24^, 2024 | N/A | N/A | N/A | N/A | N/A | Patients were followed until a diagnosis of ileus or intestinal obstruction, death, emigration, or end of follow-up. |
| Gorelik^42^, 2024 | Obesity: BMI ≥30 and non-obese (BMI < 30). | IBD-related hospitalization was considered if longer than 1 day and had an IBD-related diagnosis at discharge. | Abdominal/  perianal  surgery | Steroid dependency: as at least 4 dispensations of systemic corticosteroids within 1 year, or a consecutive treatment duration of at least 90 days. | Initiation of a biologic or small molecule therapy, not used in a previous study period, or a switch to a different class. | Composite of steroid dependency, IBD treatment escalation, IBD-related hospitalization, abdominal/perianal  surgery, or death. |
| St-Pierre^43^, 2024 | N/A | N/A | N/A | N/A | N/A | Efficacy of GLP1-RAs in non-diabetic patients with IBD, for which the indication for treatment was weight loss. |
| Levine^22^, 2024 | N/A | IBD-related. | IBD-related. | Corticosteroids use. | Escalation from nonadvanced IBD therapy to advanced IBD therapy OR change within advanced IBD therapy. | IBD exacerbation in the 12-month post–GLP-1 receptor agonist prescription. IBD exacerbation was defined as any of the following: (1) IBD-related surgery; (2) IBD-related hospitalization; (3) corticosteroid prescription, and (4) escalation from nonadvanced IBD therapy to advanced IBD therapy OR change within advanced IBD therapy. |
| Ramos Belinchón^27^, 2024 | BMI ≥ 30 | N/A | N/A | N/A | N/A | Percentage of change in  body weight from baseline to six months and a weight re  duction of 5 % or more at six months. |
| Desai^23^, 2024 (1) | Overweight  (BMI: 25–29.9), obesity (30–39.9) and morbid obesity (≥40 | Hospitalization requiring intravenous methylprednisolone | IBD-related surgery within 3 years in the GLP- 1RA and control cohorts. IBD-related surgery for the UC cohort was total colectomy while for CD was partial or total colectomy and/or enterectomy. | Patients with oral steroid use were  identified using the Rxnorm code for Prednisone. | Advanced  therapy initiation included patients who had Rxnorm codes for  infliximab, adalimumab, golimumab, certolizumab, vedolizumab,  ustekinumab, tofacitinib, upadacitinib, ozanimod or risankizumab. | Primary outcome was hospitalization requiring intravenous steroids and  IBD- related surgery within 3 years |
| Desai^44^, 2024 (2) | BMI ≥30 kg/m2 plus an RxNorm code for semaglutide  (1991302) | (1) Hospitalization requiring intravenous steroid use.  (2) Any-cause hospitalization  Patients who required hospitalization were identified using the Current  Procedural Terminology (CPT) code for “Hospital  Inpatient Services” (1013659) or “Inpatient Encounter” visit. | IBD-related  surgery included patients requiring partial colectomy,  total colectomy, and/or enterectomy. | Oral steroid use. | Initiation of advanced therapies in bio-naïve patients. | Mean total body weight change between 6 and 15 months from initiation of semaglutide compared with baseline between the 2 cohorts. |
| Sehgal^28^, 2024 | N/A | N/A | N/A | N/A | N/A | Continuation of GLP1-RA at the end of the 3-month interval. |
| Villumsen^45^, 2021 | N/A | IBD-related inpatient hospitalizations | IBD related major surgery defined as colectomies, resections, and other unspecified major intestinal operations (yes, no), and IBD-related minor surgery defined as intra-abdominal abscess drainage, fistula surgery, and surgery for perianal complications (0, 1 2, >2) | Redemption of any oral corticosteroid during follow-up. | TNF-a-inhibitors was  obtained through the Danish National Patient Registry with treatment classification codes for infliximab, adalimumab, and golimumab, and redemptions in the Danish National Prescription Registry  for adalimumab. | Composite of the need for oral corticosteroid treatment, need for TNF-a-inhibitor treatment, IBD-related hospitalization, or IBD-related major surgery. |

**
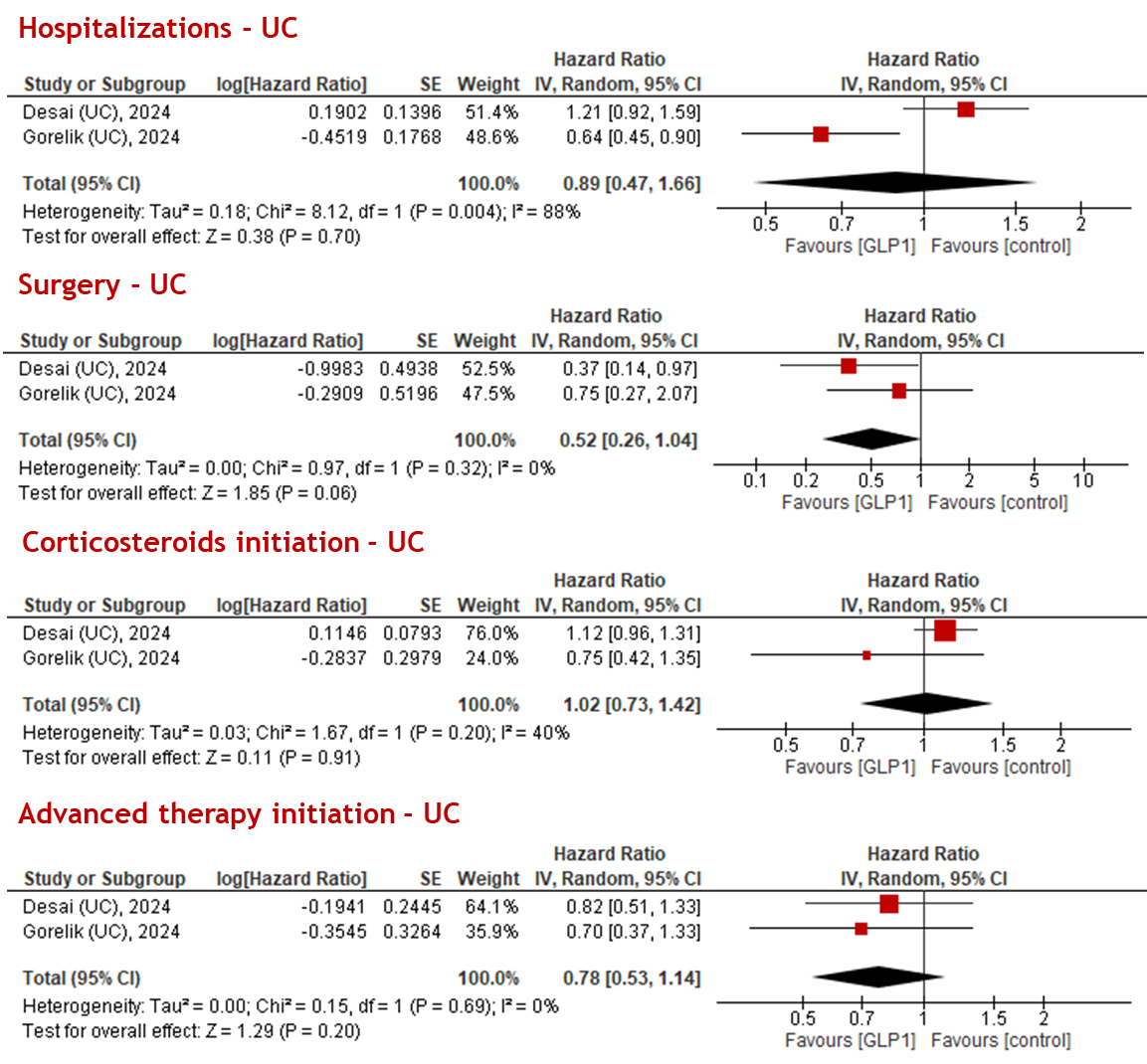
**

**Supplement 3.** Meta-analysis of different IBD-related outcomes (hospitalizations, surgery, corticosteroids initiation and advanced therapy initiation) in various studies with UC patients based on published effect sizes (hazard ratio). GLP1-RA therapy did not affect any outcome significantly different compared to controls.


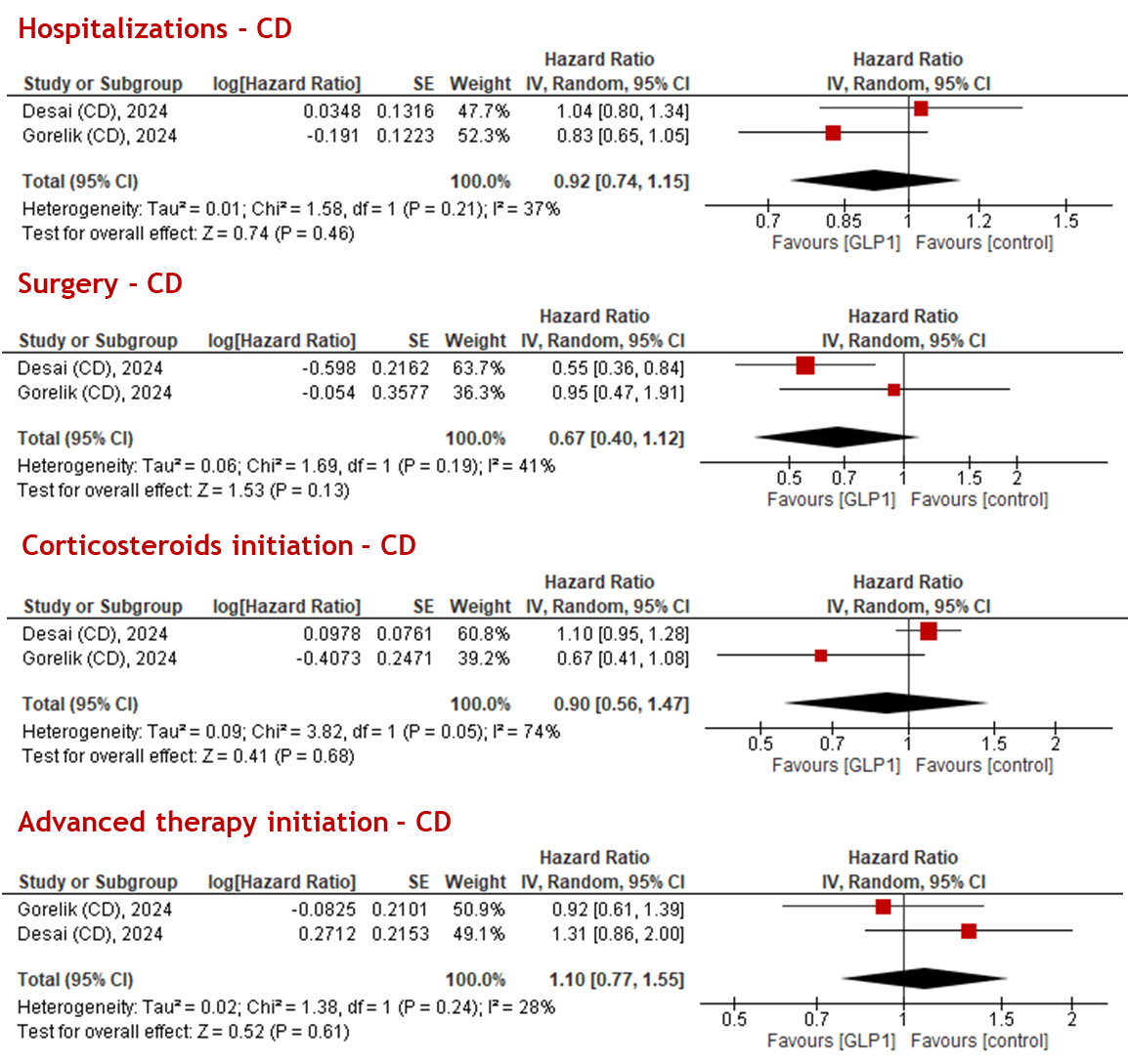


**Supplement 4.** Meta-analysis of different IBD-related outcomes (hospitalizations, surgery, corticosteroids initiation and advanced therapy initiation) in various studies with CD patients based on published effect sizes (hazard ratio). GLP1-RA therapy did not affect any outcome significantly different compared to controls.

**Supplement 5. Metabolic or chemical changes in IBD patients after GLP1 therapy.**

| Author, year | Weight change | HbA1c (%) | Creatinine | CRP | FCP |
| --- | --- | --- | --- | --- | --- |
| Clarke^14^, 2025 | Total weight loss (8.7% ± 9.0%)  BMI change -3.3±3.6  Weight loss ≥ 5% (n = 106, 60.6%)  Weight loss ≥ 10% (n = 73, 41.7%) | **N.A.** | **N.A.** | **N.A.** | **N.A.** |
| Anderson^26^, 2024 | **N.A.** | **N.A.** | **N.A.** | **Prior to GLP1:** 12.9 mg/L  **12-months post GLP1:** 6.4 mg/L  P = 0.005* | **N.A.** |
| Nielsen^24^, 2024 | **N.A.** | **N.A.** | **N.A.** | **N.A.** | **N.A.** |
| Gorelik^42^, 2024 | **N.A.** | **Baseline**  **GLP1 users:** 7.2 (IQR 6.3-8.2)  **Non-GLP1**: 6.5 (IQR 6.1-7.2) | **N.A.** | **Baseline**  **GLP1 users:** 0.9 (IQR 0.4-2.1)  **Non-GLP1**: 0.8 (IQR 0.3-2.1) | **Baseline**  **GLP1 users:** 116 (IQR 62-319)  **Non-GLP1**: 166 (IQR 70-385) |
| St-Pierre^43^, 2024 | **Baseline BMI:** 34.0 (IQR 31.0-38.2)  **Follow-up BMI:** 31.0 (IQR 29.0-36.1)  **P < 0.0001*** | **N.A.** | **N.A.** | **Baseline**: 3 mg/L (IQR 3–6.5 mg/L).  **Follow-up**: 3 mg/L, (IQR 3–6.5 mg/L)  P = 0.21 | **Low number of patients.** |
| Levine^22^, 2024 | **IBD cases (BMI)**  **Baseline:** 33.5 (29.4-37.5)  **12-months:** 31.6 (28.2-36.2)  **P < 0.01***  **Non-IBD cases (BMI)**  **Baseline:** 36.1 (31.9-41.0)  **12-months:** 34.4 (30.1-39.6)  **P < 0.01*** | **IBD cases**  **Baseline:** 6.5 (5.9-7.4)  **12-months:** 6.2 (5.4-7.0)  **P = 0.01***  **Non-IBD cases**  **Baseline:** 6.6 (5.9-8.0)  **12-months:** 6.3 (5.7-7.6)  **P = 0.03*** | **N.A.** | **Prior to GLP1:** 6.9 mg/L (3.1-11.4)  **12-months post GLP1**: 5.5 mg/L (2.8-11.6) | **N.A.** |
| Ramos Belinchón^27^, 2024 | **Baseline:** 92.2 kg (83.4-94.6)  **6-months:** 82.0 (76.6-87.7)  **P = 0.002*** | **Baseline:** 6.2% (5.5-7.8)  **6-months:** 5.9% (5.3-7.58)  P = 0.14 | **Baseline:** 6.9 mg/L (6.5-8.4)  **6-months:** 7 mg/L (6.0-9.5)  P = 0.12 | **Baseline:** 4.5 mg/L (4.0-7.8)  **6-months:** 7.0 mg/L (6.0-9.5) | **Baseline:** 41.0 µg/g (14-80)  **6-months:** 32.7 µg/g (13.8-51.0)  P = 0.74 |
| Desai^23^, 2024 (1) | **N.A.** | **N.A.** | **N.A.** | **N.A.** | **UC group**  **GLP1:** 324.7 ± 533  **Controls:** 369.7 ± 624  P = 0.67  **CD group**  **GLP1:** 260 ± 391  **Controls:** 324 ± 487  P = 0.29 |
| Desai^44^, 2024 (2) | **ΔTBW (95% CI) (pounds)**  **Semaglutide: -25**  Vs.  **Phentermine-topiramate:** -11 (P < 0.0001)  Vs.  **Bupropion-naltrexone:** - 3 (P <0.0001)  **Orlistat:** -12 (P = 0.007)  **ΔTBW (pounds) in IBD patients, different GLP1-RAs**  **Liraglutide:** -13  **Semaglutide:** -19  P = 0.04  **Tirzepatide:** -26  **Semaglutide:** -18  P = 0.01 | **N.A.** | **N.A.** | **N.A.** | **N.A.** |
| Sehgal^28^, 2024 | **Baseline:** 102 kg (SD 26.4)  **Week 12-24:** 97.6 kg (SD 28.0) | **N.A.** | **N.A.** | **Baseline:** 10.1 mg/dL (SD 16.6).  **Follow-up:** 3.0 mg/dL (SD 6.3)  P < 0.01 | **Baseline:** 824.9 µg/g (SD 2112.7)  **Week 12-24:** 234.7 µg/g (SD 530.8)  P = 0.13 |
| Villumsen^45^, 2021 | **N.A.** | **N.A.** | **N.A.** | **N.A.** | **N.A.** |

**Supplement 6.** Sub-analysis from Desai et al.^23^ for BMI (BMI ≥30, BMI < 30) in IBD patients.

| Outcomes | IBD-GLP1-RA cohort | IBD Control cohort | aHR | 95% CI | P value |
| --- | --- | --- | --- | --- | --- |
| BMI >= 30 | N = 1309 | N = 1309 |  |  |  |
| Composite outcome | 668 (51%) | 710 (54.2%) | 0.91 | 0.82-1.01 | 0.09 |
| Surgery | 47 (3.59%) | 109 (8.37%) | 0.44 | 0.31-0.61 | **<0.0001** |
| IV steroids requiring hospitalization | 339 (25.8%) | 354 (27%) | 0.98 | 0.84-1.14 | 0.84 |
| Oral steroids | 498 (38%) | 552 (42.1%) | 0.88 | 0.78-0.99 | 0.04 |
| New advanced therapy | 49 (4.74%) | 53 (5.02%) | 0.98 | 0.66-1.45 | 0.93 |
| Any steroid | 650 (49.6%) | 669 (51.1%) | 0.96 | 0.86-1.07 | 0.46 |
|  |  |  |  |  |  |
| BMI < 30 | N = 379 | N = 379 |  |  |  |
| Composite outcome | 181 (47.7%) | 200 (52.7%) | 0.86 | 0.70-1.05 | 0.15 |
| Surgery | 17 (4.48%) | 31 (8.17%) | 0.55 | 0.30-0.99 | **0.04** |
| IV steroids requiring hospitalization | 97 (25.5%) | 104 (27.4%) | 0.93 | 0.71-1.23 | 0.64 |
| Oral steroids | 129 (34%) | 153 (40.3%) | 0.79 | 0.63-1.007 | 0.05 |
| New advanced therapy | 16 (5.6%) | 16 (5.3%) | 1.05 | 0.52-2.11 | 0.87 |
| Any steroid | 175 (46.1%) | 190 (50.1%) | 0.88 | 0.71-1.08 | 0.23 |

**Supplement 7.** Sub-analysis from Desai et al.^23^ for BMI (BMI ≥30, BMI < 30) in UC patients.

| Outcomes | UC-GLP1-RA cohort | UC Control cohort | aHR | 95% CI | P value |
| --- | --- | --- | --- | --- | --- |
| BMI >= 30 | N = 617 | N = 617 |  |  |  |
|  |  |  |  |  |  |
| Composite outcome | 303 (49.1%) | 299 (48.4%) | 1.04 | 0.89-1.23 | 0.56 |
| Surgery | <10 | 14 (2.26%) | 0.21 | 0.06-0.76 | **0.008** |
| IV steroids requiring hospitalization | 153 (24.6%) | 151 (24.4%) | 1.01 | 0.78-1.31 | 0.89 |
| Oral steroids | 230 (37.2%) | 244 (39.5%) | 0.95 | 0.79-1.14 | 0.59 |
| New advanced therapy | 21 (3.75%) | 14 (2.50%) | 1.58 | 0.80-3.11 | 0.17 |
| Any steroid | 301 (48.7%) | 293 (47.4%) | 1.06 | 0.91-1.25 | 0.41 |
|  |  |  |  |  |  |
| BMI < 30 | N = 259 | N = 259 |  |  |  |
| Composite outcome | 119 (45.9%) | 138 (53.2%) | 0.82 | 0.64-1.05 | 0.12 |
| Surgery | <10 | <10 | - | - | - |
| IV steroids requiring hospitalization | 64 (24.7%) | 69 (26.6%) | 0.99 | 0.71-1.40 | 0.99 |
| Oral steroids | 81 (31.2%) | 111 (42.8%) | 0.66 | 0.50-0.89 | **0.005** |
| New advanced therapy | 11 (5.36%) | 15 (7.28%) | 0.76 | 0.35-1.67 | 0.50 |
| Any steroid | 117 (45.1%) | 134 (51.7%) | 0.84 | 0.65-1.07 | 0.17 |

**Supplement 8.** Sub-analysis from Desai et al.^23^ for BMI (BMI ≥30, BMI < 30) in CD patients.

| Outcomes | CD-GLP1-RA cohort | CD Control cohort | aHR | 95% CI | P value |
| --- | --- | --- | --- | --- | --- |
| BMI >= 30 | N = 483 | N = 483 |  |  |  |
| Composite outcome | 228 (47.2%) | 257 (53.2%) | 0.81 | 0.68-0.97 | **0.02** |
| Surgery | 13 (2.69%) | 30 (6.2%) | 0.44 | 0.23-0.85 | **0.01** |
| IV steroids requiring hospitalization | 109 (22.5%) | 142 (29.4%) | 0.70 | 0.59-0.97 | **0.03** |
| Oral steroids | 182 (37.6%) | 199 (41.2%) | 0.87 | 0.71-1.07 | 0.19 |
| New advanced therapy | 20 (5.4%) | 21 (5.3%) | 1.05 | 0.56-1.93 | 0.87 |
| Any steroid | 226 (46.7%) | 251 (51.9%) | 0.84 | 0.70-1.00 | 0.05 |
|  |  |  |  |  |  |
| BMI < 30 | N = 193 | N = 193 |  |  |  |
| Composite outcome | 91 (47.1%) | 119 (61.6%) | 0.65 | 0.49-0.85 | **0.002** |
| Surgery | 11 (5.6%) | 17 (8.8%) | 0.65 | 0.30-1.40 | 0.27 |
| IV steroids requiring hospitalization | 49 (25.3%) | 71 (36.7%) | 0.65 | 0.45-0.93 | **0.02** |
| Oral steroids | 68 (35.2%) | 88 (45.5%) | 0.70 | 0.51-0.96 | **0.02** |
| New advanced therapy | 10 (8.0%) | 13 (9.7%) | 0.58 | 0.23-1.46 | 0.24 |
| Any steroid | 87 (45.0%) | 114 (59.0%) | 0.65 | 0.49-0.86 | **0.002** |

**Supplement 9.** Sub-analysis from Gorelik et al.^42^ for BMI (BMI ≥30, BMI < 30).

|  | BMI≥30 |  |  | BMI<30 |  |  |
| --- | --- | --- | --- | --- | --- | --- |
|  | All (n=1475) | UC (n=720) | CD (n=755) | All (n=2120) | UC (n=1084) | CD (n=1036) |
| Composite event rate (per 1000 patient years) | 310 (299, 321) | 287 (272, 302) | 335 (318, 352) | 320 (311, 329) | 292 (380, 305) | 350 (336, 364) |
| Hospitalization event rate (per 1000 patient years) | 170 (162, 179) | 162 (151, 173) | 179 (167, 191) | 174 (167, 181) | 162 (153, 172) | 186 (176, 197) |
| Surgery event rate (per 1000 patient years) | 14 (12, 17) | 11 (9, 15) | 17 (13, 21) | 13 (12, 15) | 10 (8, 12) | 17 (14, 21) |
| Steroid dependence event rate (per 1000 patient years) | 128 (121, 135) | 126 (116, 136) | 130 (120, 141) | 145 (139, 151) | 132 (124, 141) | 158 (149, 168) |
| Treatment change event rate (per 1000 patient years) | 41 (37, 45) | 22 (18, 27) | 61 (54, 68) | 35 (32, 38) | 26 (22, 30) | 44 (40, 50) |
| GLP-1 HR Composite outcome | 0.62 (0.50, 0.77) | 0.63 (0.45, 0.89) | 0.60 (0.46, 0.79) | 0.93 (0.67, 1.31) | 0.77 (0.37, 1.61) | 1.05 (0.73, 1.52) |
| GLP-1 HR Hospitalization outcome | 0.70 (0.55, 0.89) | 0.66 (0.44, 0.98) | 0.72 (0.53, 0.97) | 0.77 (0.55, 1.08) | 0.49 (0.21, 1.10) | 0.94 (0.66, 1.35) |
| GLP-1 HR Surgery outcome | 0.50 (0.22, 1.10) | 0.45 (0.12, 1.64) | 0.54 (0.21, 1.42) | 1.54 (0.70, 3.38) | 1.90 (0.50, 7.30) | 1.45 (0.54, 3.90) |
| GLP-1 HR Steroid dependence outcome | 0.50 (0.32, 0.78) | 0.62 (0.33, 1.17) | 0.38 (0.21, 0.69) | 1.07 (0.58, 1.95) | 0.97 (0.25, 3.82) | 1.14 (0.60, 2.17) |
| GLP-1 HR Treatment change outcome | 0.55 (0.34, 0.91) | 0.49 (0.20, 1.21) | 0.61 (0.33, 1.11) | 1.38 (0.88, 2.18) | 1.11 (0.48, 2.57) | 1.52 (0.88, 2.61) |

**Supplement 10.** Additional data from Villumsen et al.^45^ for different clinical outcomes (hospitalizations, surgery, steroid initiation and advanced therapy initiation) in IBD patients.

|  | GLP-1 users | | Non-users | |
| --- | --- | --- | --- | --- |
|  | **Events** | **Total** | **Events** | **Total** |
| Hospitalization | 178 | 981 | 1445 | 2767 |
| Surgery | 97 | 981 | 593 | 2767 |
| Steroid initiation | 133 | 892 | 1238 | 2516 |
| TNFi initiation | 29 | 978 | 213 | 2759 |

**Supplement 11**. Weight loss in IBD patients treated with GLP1-RAs by Clarke et al.^14^

| GLP-1 Therapy | Overall Baseline Weight mean, sd (kg)  (N=272) | Baseline Weight in those with 12-month data mean, sd (kg)  (N=175) | 12-month Weight, mean, sd (kg) | Weight Difference mean, sd (kg) | TWL% mean, sd |
| --- | --- | --- | --- | --- | --- |
| Semaglutide  (N=75) | 102.0 (20.98) | 102.52 (19.75) | 90.66 (20.16) | 11.86 (9.56) | 11.55 (8.42) |
| Liraglutide  (N=34) | 105.6 (19.64) | 111.04 (20.45) | 102.55 (22.03) | 8.49 (9.82) | 7.74 (8.56) |
| Tirzepatide  (N=13) | 113 (26.01) | 118.9 (22.7) | 104.7 (28.94) | 14.18 (16.64) | 12.39 (13.73) |
| Dulaglutide  (N=51) | 101.2 (22.31) | 103.28 (23.45) | 97.41 (21.61) | 4.97 (7.01) | 4.56 (6.77) |
| Exenatide  (N=1) | 111.8 (6.79) | 116.6 (-) | 112 (-) | 4.6 (-) | 3.95 |

**Supplement 12**. Weight loss in IBD patients treated with GLP1-RAs by Sehgal et al.^28^

| GLP-1RA | weight (kg) | SD | weight 2 (kg) at 3-6 months post initiation of GLP-1RA | SD | change in weight (kg) | percent change in weight |
| --- | --- | --- | --- | --- | --- | --- |
| Dulaglutide | 97.4 | 24.2 | 93.7 | 24.1 | 3.7 | 1.7 |
| Semaglutide | 101.0 | 26.0 | 94.7 | 23.5 | 6.3 | 2.8 |
| Liraglutide | 110.8 | 30.2 | 105.8 | 31.6 | 5.0 | 2.0 |
| Exenatide | 110.7 | 16.5 | 109.0 | 17.6 | 1.7 | 0.7 |
| Tirzepatide | 112.2 | 21.3 | 107.7 | 19.9 | 4.5 | 1.8 |

**Supplement 13.** Weight loss in IBD patients treated with GLP1-RAs by Anderson et al.^26^

| GLP1-RA | Baseline mean (kg) | SD (kg) | Follow-up mean (kg) | Follow-up SD (kg) |
| --- | --- | --- | --- | --- |
| Dulaglutide | 110.08 | 23.25 | 110.04 | 22.84 |
| Exenatide | 109.65 | 40.80 | 115.14 | 20.63 |
| Liraglutide | 94.85 | 26.08 | 100.47 | 30.75 |
| Semaglutide | 113.48 | 23.40 | 110.01 | 21.99 |

**Supplement 14.** Weight loss in IBD patients treated with GLP1-RAs by Desai et al.^44^

|  | Baseline in kg | After 6-15 mo in kg |
| --- | --- | --- |
| IBD Tirzepatide | 109.3 +/- 25.9 | 97.5 +/- 26.8 |
| IBD Liraglutide | 102.5 +/- 20.4 | 96.6 +/- 24.0 |

**Supplement 15. Research checklist**. PRISMA Checklist.^20^

| **Section and Topic** | **Item #** | **Checklist item** | **Location where item is reported** |
| --- | --- | --- | --- |
| **TITLE** | | |  |
| Title | 1 | Identify the report as a systematic review. | Page 1 |
| **ABSTRACT** | | |  |
| Abstract | 2 | See the PRISMA 2020 for Abstracts checklist. | Page 2 |
| **INTRODUCTION** | | |  |
| Rationale | 3 | Describe the rationale for the review in the context of existing knowledge. | Page 5 |
| Objectives | 4 | Provide an explicit statement of the objective(s) or question(s) the review addresses. | Page 5 |
| **METHODS** | | |  |
| Eligibility criteria | 5 | Specify the inclusion and exclusion criteria for the review and how studies were grouped for the syntheses. | Page 5-6 |
| Information sources | 6 | Specify all databases, registers, websites, organisations, reference lists and other sources searched or consulted to identify studies. Specify the date when each source was last searched or consulted. | Page 6 |
| Search strategy | 7 | Present the full search strategies for all databases, registers and websites, including any filters and limits used. | Page 6 |
| Selection process | 8 | Specify the methods used to decide whether a study met the inclusion criteria of the review, including how many reviewers screened each record and each report retrieved, whether they worked independently, and if applicable, details of automation tools used in the process. | Page 6-7 |
| Data collection process | 9 | Specify the methods used to collect data from reports, including how many reviewers collected data from each report, whether they worked independently, any processes for obtaining or confirming data from study investigators, and if applicable, details of automation tools used in the process. | Page 6-7 |
| Data items | 10a | List and define all outcomes for which data were sought. Specify whether all results that were compatible with each outcome domain in each study were sought (e.g. for all measures, time points, analyses), and if not, the methods used to decide which results to collect. | Page 6-7 |
|  | 10b | List and define all other variables for which data were sought (e.g. participant and intervention characteristics, funding sources). Describe any assumptions made about any missing or unclear information. | Page 6-7 |
| Study risk of bias assessment | 11 | Specify the methods used to assess risk of bias in the included studies, including details of the tool(s) used, how many reviewers assessed each study and whether they worked independently, and if applicable, details of automation tools used in the process. | Page 6-7 |
| Effect measures | 12 | Specify for each outcome the effect measure(s) (e.g. risk ratio, mean difference) used in the synthesis or presentation of results. | Page 6-7 |
| Synthesis methods | 13a | Describe the processes used to decide which studies were eligible for each synthesis (e.g. tabulating the study intervention characteristics and comparing against the planned groups for each synthesis (item #5)). | Page 6-7 |
|  | 13b | Describe any methods required to prepare the data for presentation or synthesis, such as handling of missing summary statistics, or data conversions. | Page 6-7 |
|  | 13c | Describe any methods used to tabulate or visually display results of individual studies and syntheses. | Page 6-7 |
|  | 13d | Describe any methods used to synthesize results and provide a rationale for the choice(s). If meta-analysis was performed, describe the model(s), method(s) to identify the presence and extent of statistical heterogeneity, and software package(s) used. | Page 6-7 |
|  | 13e | Describe any methods used to explore possible causes of heterogeneity among study results (e.g. subgroup analysis, meta-regression). | Page 6-7 |
|  | 13f | Describe any sensitivity analyses conducted to assess robustness of the synthesized results. | Page 6-7 |
| Reporting bias assessment | 14 | Describe any methods used to assess risk of bias due to missing results in a synthesis (arising from reporting biases). | - |
| Certainty assessment | 15 | Describe any methods used to assess certainty (or confidence) in the body of evidence for an outcome. | Page 6-7 |
| **RESULTS** | | |  |
| Study selection | 16a | Describe the results of the search and selection process, from the number of records identified in the search to the number of studies included in the review, ideally using a flow diagram. | Page 7 |
|  | 16b | Cite studies that might appear to meet the inclusion criteria, but which were excluded, and explain why they were excluded. | Figure 1 |
| Study characteristics | 17 | Cite each included study and present its characteristics. | Table 2 |
| Risk of bias in studies | 18 | Present assessments of risk of bias for each included study. | Table 1 |
| Results of individual studies | 19 | For all outcomes, present, for each study: (a) summary statistics for each group (where appropriate) and (b) an effect estimate and its precision (e.g. confidence/credible interval), ideally using structured tables or plots. | Table 2-4 |
| Results of syntheses | 20a | For each synthesis, briefly summarise the characteristics and risk of bias among contributing studies. | Table 1 |
|  | 20b | Present results of all statistical syntheses conducted. If meta-analysis was done, present for each the summary estimate and its precision (e.g. confidence/credible interval) and measures of statistical heterogeneity. If comparing groups, describe the direction of the effect. | Figure 2-5 |
|  | 20c | Present results of all investigations of possible causes of heterogeneity among study results. | Page 7-10 |
|  | 20d | Present results of all sensitivity analyses conducted to assess the robustness of the synthesized results. | Page 7-10 |
| Reporting biases | 21 | Present assessments of risk of bias due to missing results (arising from reporting biases) for each synthesis assessed. | Table 1 |
| Certainty of evidence | 22 | Present assessments of certainty (or confidence) in the body of evidence for each outcome assessed. | Table 1 |
| **DISCUSSION** | | |  |
| Discussion | 23a | Provide a general interpretation of the results in the context of other evidence. | Page 11-13 |
|  | 23b | Discuss any limitations of the evidence included in the review. | Page 11-13 |
|  | 23c | Discuss any limitations of the review processes used. | Page 11-13 |
|  | 23d | Discuss implications of the results for practice, policy, and future research. | Page 11-13 |
| **OTHER INFORMATION** | | |  |
| Registration and protocol | 24a | Provide registration information for the review, including register name and registration number, or state that the review was not registered. | Page 5 |
|  | 24b | Indicate where the review protocol can be accessed, or state that a protocol was not prepared. | Page 5 |
|  | 24c | Describe and explain any amendments to information provided at registration or in the protocol. | Page 5 |
| Support | 25 | Describe sources of financial or non-financial support for the review, and the role of the funders or sponsors in the review. | Page 1 |
| Competing interests | 26 | Declare any competing interests of review authors. | Page 1 |
| Availability of data, code and other materials | 27 | Report which of the following are publicly available and where they can be found: template data collection forms; data extracted from included studies; data used for all analyses; analytic code; any other materials used in the review. | Supplement 6-10 |
